# Clinical Diagnosis of the Dampness and Mold Hypersensitivity Syndrome: Review of the Literature and Suggested Diagnostic Criteria

**DOI:** 10.3389/fimmu.2017.00951

**Published:** 2017-08-10

**Authors:** Ville Valtonen

**Affiliations:** ^1^Division of Infectious Diseases, Department of Medicine, Helsinki University Central Hospital, Helsinki, Finland

**Keywords:** mold, dampness, multiple chemical sensitivity, sick building syndrome, clinical classification, HLA genes

## Abstract

A great variety of non-specific symptoms may occur in patients living or working in moisture-damaged buildings. In the beginning, these symptoms are usually reversible, mild, and present irritation of mucosa and increased morbidity due to respiratory tract infections and asthma-like symptoms. Later, the disease may become chronic and a patient is referred to a doctor where the assessment of dampness and mold hypersensitivity syndrome (DMHS) often presents diagnostic challenges. Currently, unanimously accepted laboratory tests are not yet available. Therefore, the diagnosis of DMHS is clinical and is based on the patient’s history and careful examination. In this publication, I reviewed contemporary knowledge on clinical presentations, laboratory methods, and clinical assessment of DMHS. From the literature, I have not found any proposed diagnostic clinical criteria. Therefore, I propose five clinical criteria to diagnose DMHS: (1) the history of mold exposure in water-damaged buildings, (2) increased morbidity to due infections, (3) sick building syndrome, (4) multiple chemical sensitivity, and (5) enhanced scent sensitivity. If all the five criteria are met, the patient has a very probable DMHS. To resolve the current problems in assigning correct DMHS diagnosis, we also need novel assays to estimate potential risks of developing DMHS.

## Introduction

There is growing variety of clinical symptoms related to the poor indoor air quality, especially in water-damaged buildings infested by hazardous microbiota (1). As a rule, dampness and mold hypersensitivity syndrome (DMHS), as we call this clinical condition here presents with signs of irritation of the respiratory tract and/or the eyes. Subsequently, the patient may experience recurrent sinusitis or bronchitis and neurological manifestations, such as headaches, nausea, and unexplained fatigue. Some may develop rheumatic symptoms resembling fibromyalgia or neurological symptoms may progress into pains and/or numbness in the legs and arms and the so-called brain fog (2–4). Some patients develop newly onset asthma, or may present asthma-like conditions, such as dyspnea, burning sensation in the respiratory tract, and productive or non-productive cough.

## The Natural Course of the DMHS

There is a huge variation in the time preceding the onset of symptoms in individuals who lived or worked in environment contaminated with moisture-related microbiota. The time interval can vary from a few months to several years. It is not known which factors may contribute to individual susceptibility to DMHS. It seems that cumulative exposure time during an individual’s lifespan when he/she is exposed to dampness microbiota may be a decisive factor. The age of the person when he/she will be exposure to moisture microbiota for the first time may be another critical component. One may speculate that there are several genetic factors, some of which are protective and some are permissive, that might contribute to the development of DMHS.

As a rule, DMHS begins with symptoms of irritation in eyes, nose, and respiratory tract. The eyes are itchy and reddened, the nose is blocked, sneezing, and cough are common symptoms at the beginning. An important hallmark of DMHS, as in many other diseases associated with poor indoor air quality, is the so-called sick building syndrome (SBS) (5). This syndrome means that a patient experiences aggravation or onset of symptoms when entering a certain water-damaged building. However, when he/she leaves the building the symptoms may be relieved or disappear completely. In the early phase of DMHS, the symptoms of SBS may disappear totally when he/she can avoid staying in the building for 1 or 2 days but the time period for total recovery seems to become prolonged with each new exposure until, finally, the symptoms will become irreversible even though the patient no longer visits the water-damaged building. Some unknown factors seem to trigger disease chronic course.

Dampness and mold hypersensitivity syndrome patients have increased morbidity rate due to infections of respiratory tract, e.g., recurrent sinusitis, bronchitis even pneumonias when the exposure to moldy environment continues. The disease seems to progresses from irritation to recurrent infections, such as tonsillitis, reactivations of herpes simplex virus (HSV1 and HSV2), recurrent urinary tract, skin infections, etc. Patients may experience also episodes of mild, prolonged fever and fatigue and a minority of them may develop the so-called chronic fatigue syndrome (6, 7).

Patients may complain muscle and joint pain resembling fibromyalgia. Furthermore, some other rheumatic manifestations have rarely been described in patients with DMHS (8, 9). Some patients develop functional central nervous system symptoms that are often called “brain fog” (2–4). These patients have impaired cognition, inability to concentrate, and problems with both short- and long-term memories. Occasional headaches and dizziness are also common in “brain fog.” Peripheral neurological manifestations, such as transient pains and numbness in the legs and/or arms are also reported by DMHS patients.

Dyspnea, burning pain, and irritation in the respiratory tract are very common although the variations in the peak expiratory flow (PEF) value do not fulfill classic asthma criteria. Instead, asthma-like dyspnea with mildly lowered PEF value variation that does not meet the diagnostic criteria of asthma is more common. However, the risk to develop unambiguous asthma is increased if the exposure to dampness microbiota continues (10). Abdominal symptoms related to DMHS are indistinguishable from irritable colon. Many different types of skin rashes are also common, even vasculitis-like lesions may occasionally occur (11).

According to my clinical experience (more than 1,000 DMHS patients), approximately every second DMHS patient will, finally, develop multiple chemical sensitivity (MCS) or chemical intolerance syndrome. MCS is a condition when a person experiences a complex array of recurrent unspecific symptoms attributable to low dosages of chemicals that are well tolerated by most people (11). Although many biomarkers of inflammation can be detected, there is no single-specific diagnostic laboratory test to diagnose MCS at the moment (12). The diagnosis of MCS can be only established by questionnaire and applying varying clinical definitions (13–15).

As far as I am aware, at the moment, there are no reliable epidemiological studies that would link mold-related disease to MCS. The incidence of this co-morbidity seems not yet to be reported in the literature. However, there are many symptoms that are common in both conditions, such as asthma, hay fever, allergic, and rheumatic symptoms (11, 15). As a rule, MCS seems to develop after the development of the so-called SBS but occasionally MCS may occur even without documented exposure to dampness microbiota or any history of preceding SBS. In my clinical practice to diagnose MCS, I used criteria described in the Table 1.

**Table 1:** Questionnaire to the exposed patients.

The patient is asked whether the following chemicals have explicitly an irritation action on them: Perfumes Deodorants, shaving lotion Detergents Tobacco smoke or other heavy smoke Fresh printed matters like newspapers Paints, varnishes, glues Hairdresser’s products Different dusts, especially street dust Exhaust fumes, gasoline, oil, other traffic fumes Windshield detergent Formaldehyde or some other known chemical Spices or some other food products

*If four or more chemicals, at least in four of the abovementioned groups, explicitly irritate, the diagnosis of multiple chemical sensitivity is probable*.

Electromagnetic field sensitivity (EMS) has been reported to associate strongly with chemical sensitivity (16). According to my clinical experience, DMHS occurs first, then MCS may develop in approximately half of those patients, and finally approximately one quarter of the DMHS patients will develop EMS. Only very rare EMS will develop without DMHS or without MCS.

## Serological Assays to Obtain Evidence of DMHS

Serology has been used for decades to support the diagnosis of mold-related disease. IgG and IgE responses have been most extensively studied, although there are clinical reports of a lack of any association between IgG antibodies and exposure to molds (17). Also, IgE-mediated sensitization and skin prick positivity for molds are rare (18). However, by using commercial antigens derived from *Penicillium notatum, Aspergillus niger*, and *Stachybotrys chartarum*, specific IgG-, IgM-, and IgA-class antibodies were elevated in patients compared to the controls (*n* = 500) (19). On the other hand, it was reported that clinical symptoms did not always correlate with the mold antibody levels (20). There are promising results from mold-specific saliva immunoglobulins against mycotoxin structures in a study that compared their levels in exposed individuals and controls (21). However, the authors noted that genetic differences in cytochrome P450 enzymes activities or glutathione *S*-transferase might be responsible for an individual’s vulnerability to disease (20). This suggests that in DMHS immunological response is multifactorial and explains the difficulty in exploiting serology for diagnosis.

The use of serology to support diagnosis of DMHS is problematic for several reasons. First, mold infestation is a dynamic ecological microenvironment where the relative quantities of different species may vary at different time points. This ecological system may contain not only fungi but also Gram-positive and Gram-negative bacteria. In addition, bioorganic compounds emitted from the decay and breakdown of supporting building material may contribute to the immunological response. Second, it is well known that many highly organized microbiological species can change their surface antigens to ensure adaptation and escape inactivation by the host’s immune system. For example, *Borrelia* species and parasites such as *Trypanosoma cruzi* or *Plasmodium* species have been demonstrated to modify their antigenic structures during infection (22), and several studies have revealed how the secretion of fungal antigens becomes altered in various conditions (23). These factors represent one of the greatest challenges in the development of mold-specific diagnostics. For this reason, it would be preferable that the tested antigens should be prepared from the suspected buildings (20), but this is unpractical in clinical settings. Third, the immunological insult from environmental molds may cause either activation or deprivation of the host immunologic system (24–26). It is especially notable that mycotoxins such as gliotoxin can inhibit the activity of antigen-presenting cells and limit the amount and functions of monocytes and simply kill immune cells and, thus, disarm the body’s immune response (25). Furthermore, decreased immunoglobulin production may be observed in some of heavily exposed individuals. It would be of interest to study whether or not the most potent mycotoxin-producing strains are responsible for the reduction of antibody production. Therefore, it seems apparent that novel alternative to conventional serology diagnostic approaches are required.

## Presentation of Clinical Diagnostic Criteria for DMHS

It is a clinical reality of today that there are no accepted diagnostic criteria for DMHS. Here, on the basis of my clinical expertise, I suggest criteria for consideration by international medical community, Table 2.

**Table 2:** Clinical criteria for dampness and mold hypersensitivity syndrome (DMHS).

If all the five criteria are met, the patient has very probably DMHS; if four to three criteria are met the diagnosis of DMHS is probable; and if two criteria are met and typical clinical symptoms, the diagnosis of DMHS is possible: History of mold exposure in water-damaged buildings with or without any symptoms. Increased morbidity due to infections. This is an early stage of the disease. Suffering the so-called sick building syndrome. That means that a person may feel unwell when entering a water-damaged building but the symptoms relieve or disappear when being outside the problematic building from 1 to 2 days. Development of the multiple chemical sensitivity (see Table 1). Increased scent sensitivity compared to his/her healthy stage. The patient may report ability to smell moldy odor, e.g., from clothes of a nearby standing person.

*The first criterion*: there should be an evidence of periods of patient being exposed to moisture microbes during his/her lifetime even without symptoms, i.e., when he/she has been living or working in water-damaged houses. Patients can recall if they have been living or working in the houses with leaking roofs or windows such that rain can enter or there has been flooding, i.e., conditions suitable for the growth of molds. Visible mold or mold odor in indoor air are also probable indicators for the presence of moisture microbiota even without microbiological culture confirmation. There is a reasonable correlation between the odor of molds in indoor air and quantitatively measured microbial exposure in homes (27). Importantly, microbiological culture reports unavailability by the time of the patients’ visit should not delay their medical consultation.

*The second criterion* is increased morbidity due to infectious diseases that are observed in a previously healthy person. Small children likewise adults or even pets may present with increased rate of, e.g., tonsillitis, bronchitis, skin and eye infections, and sinusitis. Especially, when a person might have more than three sinusitis/year, the doctor might start to suspect environmental factors. In the beginning, the patients or the guardians of small children may not associate the high rate of their visits to the doctors due to these infections. This phase is associated with the increased frequency of sick leaves. This is an early stage of DMHS, and the only way to prevent the development of the chronic course is to acknowledge the possibility of moldy environment and start timely investigations. However, this stage of the disease if often overlooked.

*The third criterion* is that the patient has a history of the so-called SBS. The patient notices that his/hers symptoms worsen when he/she enters the problematic building and conversely, the symptoms diminish when they are not in the building. If there is a clear history of leaky roofs or rain or moisture gaining or a clear odor of mold or visible molds in ceilings or walls, it is not necessary to have culture confirmation of moisture microbes. If molds and other typical moisture bacteria can be cultured from the structures of the problem building or high concentrations of volatile organic compounds can be detected in the indoor air, this raises the probability of the DMHS diagnosis.

*The forth criterion* is the development of MCS. I used self-made questionnaire (Table 1) to diagnose MCS. If the patient reacts (i.e., he/she is explicitly irritated) to four or more chemicals belonging to at least four different groups (Table 1), then the diagnosis of MCS is probable. The definition of what is interpreted as “explicitly irritates” is important, because it has an effect on the incidence of MCS at the population level. If the patient experiences such symptoms as nausea, headache, cough, or dyspnea, or the patient has to distance him/herself e.g., from a person using deodorants, this means indication for “explicitly irritation.”

And lastly, *the fifth criterion* is an enhanced sensitivity to odors, especially the odor of molds. For example, if the patient can smell the odor of molds from the clothes of a nearby person, this can be viewed as the positive fifth criterion.

The presence of all the five criteria (Table 2) designates advanced stage of DMHS that have been lasted for at least many months or even years. In this situation, we have probably lost the time window of opportunities to completely revert the disease. The first limitation of this approach is that it will not pick up persons at the early stages of the disease and, second, that it cannot be used in very young children because the questionnaire is not applicable.

If a person has all the five of the abovementioned criteria, then the diagnosis of DMHS is *very probable*. The presence of the four to three positive criteria means *probable* DMHS and the two positive criteria means *possible* DMHS.

## Assays of Mycotoxin and Neurological Symptoms

In invasive aspergillosis, the diagnosis often relies on methods detecting either living fungi in blood or deep tissue specimens, or specific antibodies or fungal antigens (23) or DNA. As a novel approach, one may mention measurements of fungal secondary metabolite signatures from exhaled breath (28). An indoor toxicity method (29) is a novel adjunct to diagnostics. Assays to detect mycotoxins in serum (30) and the detection of the excreted mycotoxins (e.g., urine, saliva) (31–34) may be specific diagnostic tests to detect DMHS. The test should be also sensitive and robust to be incorporated into diagnostic criteria.

One potential way to improve diagnostics would be the assays originally designed for neurologic patients with e.g., polyneuropathies to detect antibodies against neural structures, such as gangliosides, myelin-associated glycoprotein and chondroitin sulfate, and so on (35). On the other hand, it is known that low titer levels of antinuclear antibodies, rheumatoid factors, and other autoantibodies are non-specific and can be detected in many chronic infections or even aging, or neuropathies of autoimmune origin (36).

## Novel Laboratory Methods to Find Those Who are at Risk to Develop DMHS

The major histocompatibility complex (MHC), located on chromosome 6 p-arm 21.3, and the genes in the HLA region are more important determinants of autoimmune or inflammatory disorders than any other region in the human genome (37). Therefore, the immunogenetics possibly linked to genes in HLA area of chromosome 6 could possibly be used to assess individual risk to develop DMHS. There are a few studies examining how deficiency of the complement system would influence an individual’s susceptibility to mold exposure. Normally, there are two complement 4A and 4B genes, but the variation in the number of C4 genes is common. Less than two complement 4A or 4B genes are common in Finland (11 and 41%, respectively). Complement 4A *CTins* mutation (6%) is responsible for non-functional complement 4A gene, causing the incidence for non-functional C4A up to 17% (17). Therefore, this particular mutation might have harmful consequences to rather large sections of the Finnish population, from which 0.8/5.5 million inhabitants have been estimated to be exposed repeatedly to molds. Support for the important role of HLA genes is the fact that activated T cells (CD3^+^ CD26^+^ cells) and the class II major histocompatibility molecule MHC (CD3^+^ HLA-DR^+^) are found in over 90% of the mold exposed individuals when compared to the controls (34).

Missing HLA genes or a deficiency in function of these genes represent a further risk in those individuals who are mold exposed. Unfortunately, the genotyping of the traditional HLA genes is expensive and laborious, which may limit the feasibility of these markers (37).

## Directions for the Diagnosis and Treatment of DMHS

Sensitive and specific laboratory tests for the diagnosis of DMHS are definitely needed; however, it may turn out that expectations of clinicians to have only one test of high sensitivity and specificity are never met (38). For example, diagnosis of autoimmune diseases is based on a combination of clinical presentations supplemented by several diagnostic methods. It may be more realistic that a useful combination of different laboratory algorithms will be used in extended DMHS criteria. As explained above, serological testing has limitations due to high variability of species, antigens, and their cross-reactivities (39). Nonetheless, sensitive commercial serological IgG tests and basophil activation testing for chronic pulmonary aspergillosis (CPA) present some promise (40–42). From a clinical point of view, it is probable that there is some overlap between allergic bronchopulmonary mycosis, allergic bronchopulmonary aspergillosis, CPA, and DMHS (40), which should be taken into account when epidemiological studies on mold allergy are conducted in the future.

According to my experience, corroborated by others (43–48), successful cure can be achieved only if the treatment starts early enough, and the patient avoids problematic moisture-damaged building already when experiencing higher morbidity with infections and the SBS stage without yet an onset of MCS that may follow later. In the early stages of the disease, the patient may become completely asymptomatic as long as he/she continues avoiding re-exposures. Challenge to achieve complete cure becomes more demanding in patients with fully blown MCS. In these situations, any re-exposure may occur by chance almost anywhere any time and not necessarily in moldy environment. Patients’ symptoms could be impossible to link to any preceding exposure to water-damaged building. Thus, avoidance of moldy buildings is recommended to patients with SBS.

Empirically, the majority of DMHS patients are recommended to adhere to a low carbohydrate diet. However, I am not aware of any studies on the effect of different diets on the clinical course of DMHS. In addition, many DMHS patients consume large amounts of l-cysteine amino acid or *N*-acetylcysteine (NAC) and they report that they may feel better, especially their “brain fog” symptoms are relieved, at least to some extent. This treatment was not yet studied systematically in controlled studies. However, there are studies suggesting that NAC might be effective in patients with different neurological symptoms (49).

## Conclusion

It is undisputable that, at the moment, available laboratory methods do not fully support clinical practice. Prevention and treatment of DMHS would largely benefit from improved laboratory diagnostics because it may facilitate early intervention. Clinical criteria of DMHS are now presented for the first time. These criteria comprise five positions, and the assessment of the probability levels on the basis of the fulfillment of these criteria is discussed.

## Author Contributions

VV was responsible for the drafting and editing of the manuscript.

## Conflict of Interest Statement

The author declares that the research was conducted in the absence of any commercial or financial relationships that could be construed as a potential conflict of interest.

## References

[1] Baxi SN Portnoy JM Larenas-Linnemann D Phipatanakul W Environmental Allergens W. Exposure and health effects of fungi on humans. J Allergy Clin Immunol Pract (2016) 4(3):396–404. 10.1016/j.jaip.2016.01.008 26947460 PMC4861659

[2] Theoharides TC Stewart JM Hatziagelaki E Kolaitis G Brain “fog,” inflammation and obesity: key aspects of neuropsychiatric disorders improved by luteolin. Front Neurosci (2015) 9:225 10.3389/fnins.2015.00225 26190965 PMC4490655

[3] Empting LD. Neurologic and neuropsychiatric syndrome features of mold and mycotoxin exposure. Toxicol Ind Health (2009) 25(9–10):577–81. 10.1177/0748233709348393 19854819

[4] Kilburn KH. Neurobehavioral and pulmonary impairment in 105 adults with indoor exposure to molds compared to 100 exposed to chemicals. Toxicol Ind Health (2009) 25(9–10):681–92. 10.1177/0748233709348390 19793776

[5] Burge PS Sick building syndrome. Occup Environ Med (2004) 61(2):185–90. 10.1136/oem.2003.008813 14739390 PMC1740708

[6] Brewer JH Thrasher JD Straus DC Madison RA Hooper D. Detection of mycotoxins in patients with chronic fatigue syndrome. Toxins (2013) 5(4):605–17. 10.3390/toxins5040605 23580077 PMC3705282

[7] Morris G Berk M Walder K Maes M. The putative role of viruses, bacteria, and chronic fungal biotoxin exposure in the genesis of intractable fatigue accompanied by cognitive and physical disability. Mol Neurobiol (2016) 53(4):2550–71. 10.1007/s12035-015-9262-7 26081141

[8] Myllykangas-Luosujarvi R Seuri M Husman T Korhonen R Pakkala K Aho K. A cluster of inflammatory rheumatic diseases in a moisture-damaged office. Clin Exp Rheumatol (2002) 20(6):833–6. 12508776

[9] Luosujarvi RA Husman TM Seuri M Pietikainen MA Pollari P Pelkonen J Joint symptoms and diseases associated with moisture damage in a health center. Clin Rheumatol (2003) 22(6):381–5. 10.1007/s10067-003-0753-y 14677010

[10] Karvala K Toskala E Luukkonen R Uitti J Lappalainen S Nordman H. Prolonged exposure to damp and moldy workplaces and new-onset asthma. Int Arch Occup Environ Health (2011) 84(7):713–21. 10.1007/s00420-011-0677-9 21769455

[11] Dantoft TM Andersson L Nordin S Skovbjerg S. Chemical intolerance. Curr Rheumatol Rev (2015) 11(2):167–84. 10.2174/157339711102150702111101 26088215

[12] De Luca C Scordo MG Cesareo E Pastore S Mariani S Maiani G Biological definition of multiple chemical sensitivity from redox state and cytokine profiling and not from polymorphisms of xenobiotic-metabolizing enzymes. Toxicol Appl Pharmacol (2010) 248(3):285–92. 10.1016/j.taap.2010.04.017 20430047

[13] Cullen MR The worker with multiple chemical sensitivities: an overview. Occup Med (1987) 2(4):655–61. 3313760

[14] Multiple chemical sensitivity: a 1999 consensus. Arch Environ Health (1999) 54(3):147–9. 10444033 10.1080/00039899909602251

[15] Lacour M Zunder T Schmidtke K Vaith P Scheidt C Multiple chemical sensitivity syndrome (MCS) – suggestions for an extension of the U.S. MCS-case definition. Int J Hyg Environ Health (2005) 208(3):141–51. 10.1016/j.ijheh.2005.01.017 15971853

[16] Rea WJ History of chemical sensitivity and diagnosis. Rev Environ Health (2016) 31(3):353–61. 10.1515/reveh-2015-0021 27383867

[17] Taskinen TM Laitinen S Nevalainen A Vepsalainen A Meklin T Reiman M Immunoglobulin G antibodies to moulds in school-children from moisture problem schools. Allergy (2002) 57(1):9–16. 10.1034/j.1398-9995.2002.13154.x 11991303

[18] Reijula K Leino M Mussalo-Rauhamaa H Nikulin M Alenius H Mikkola J IgE-mediated allergy to fungal allergens in Finland with special reference to *Alternaria alternata* and *Cladosporium herbarum*. Ann Allergy Asthma Immunol (2003) 91(3):280–7. 10.1016/S1081-1206(10)63531-4 14533661

[19] Caffrey AK Lehmann MM Zickovich JM Espinosa V Shepardson KM Watschke CP IL-1alpha signaling is critical for leukocyte recruitment after pulmonary *Aspergillus fumigatus* challenge. PLoS Pathog (2015) 11(1):e1004625 10.1371/journal.ppat.1004625 25629406 PMC4309569

[20] Vojdani A Campbell AW Kashanian A Vojdani E. Antibodies against molds and mycotoxins following exposure to toxigenic fungi in a water-damaged building. Arch Environ Health (2003) 58(6):324–36. 10.1080/00039896.2003.11879143 14992307

[21] Vojdani A Kashanian A Vojdani E Campbell AW. Saliva secretory IgA antibodies against molds and mycotoxins in patients exposed to toxigenic fungi. Immunopharmacol Immunotoxicol (2003) 25(4):595–614. 10.1081/IPH-120026444 14686801

[22] Johnson PL Kochin BF Ahmed R Antia R. How do antigenically varying pathogens avoid cross-reactive responses to invariant antigens? Proc Biol Sci (2012) 279(1739):2777–85. 10.1098/rspb.2012.0005 22438498 PMC3367775

[23] Kniemeyer O Ebel F Kruger T Bacher P Scheffold A Luo T Immunoproteomics of *Aspergillus* for the development of biomarkers and immunotherapies. Proteomics Clin Appl (2016) 10(9–10):910–21. 10.1002/prca.201600053 27312145

[24] Anyanwu E Campbell AW Vojdani A Ehiri JE Akpan AI. Biochemical changes in the serum of patients with chronic toxigenic mold exposures: a risk factor for multiple renal dysfunctions. ScientificWorldJournal (2003) 3:1058–64. 10.1100/tsw.2003.92 14612611 PMC5974800

[25] Stanzani M Orciuolo E Lewis R Kontoyiannis DP Martins SL St John LS *Aspergillus fumigatus* suppresses the human cellular immune response via gliotoxin-mediated apoptosis of monocytes. Blood (2005) 105(6):2258–65. 10.1182/blood-2004-09-3421 15546954

[26] Meissonnier GM Pinton P Laffitte J Cossalter AM Gong YY Wild CP Immunotoxicity of aflatoxin B1: impairment of the cell-mediated response to vaccine antigen and modulation of cytokine expression. Toxicol Appl Pharmacol (2008) 231(2):142–9. 10.1016/j.taap.2008.04.004 18501398

[27] Reponen T Singh U Schaffer C Vesper S Johansson E Adhikari A Visually observed mold and moldy odor versus quantitatively measured microbial exposure in homes. Sci Total Environ (2010) 408(22):5565–74. 10.1016/j.scitotenv.2010.07.090 20810150 PMC2972663

[28] Koo S Thomas HR Daniels SD Lynch RC Fortier SM Shea MM A breath fungal secondary metabolite signature to diagnose invasive aspergillosis. Clin Infect Dis (2014) 59(12):1733–40. 10.1093/cid/ciu725 25342502 PMC4311178

[29] Salo J Andersson MA Mikkola R Kredics L Viljanen M Salkinoja-Salonen M, editors. Vapor as a carrier of toxicity in a health troubled building. Healthy Buildings Europe. (Vol. 2015), 2015 May; Eindhoven, The Netherlands (2015). p. 18–20.

[30] Domingo MP Colmenarejo C Martinez-Lostao L Mullbacher A Jarne C Revillo MJ Bis(methyl)gliotoxin proves to be a more stable and reliable marker for invasive aspergillosis than gliotoxin and suitable for use in diagnosis. Diagn Microbiol Infect Dis (2012) 73(1):57–64. 10.1016/j.diagmicrobio.2012.01.012 22480566

[31] Hooper DG Bolton VE Guilford FT Straus DC. Mycotoxin detection in human samples from patients exposed to environmental molds. Int J Mol Sci (2009) 10(4):1465–75. 10.3390/ijms10041465 19468319 PMC2680627

[32] Desalegn B Nanayakkara S Harada KH Hitomi T Chandrajith R Karunaratne U Mycotoxin detection in urine samples from patients with chronic kidney disease of uncertain etiology in Sri Lanka. Bull Environ Contam Toxicol (2011) 87(1):6–10. 10.1007/s00128-011-0301-4 21553028

[33] Rea WJ Didriksen N Simon TR Pan Y Fenyves EJ Griffiths B. Effects of toxic exposure to molds and mycotoxins in building-related illnesses. Arch Environ Health (2003) 58(7):399–405. 10.1080/00039896.2003.11879140 15143852

[34] Gray MR Thrasher JD Crago R Madison RA Arnold L Campbell AW Mixed mold mycotoxicosis: immunological changes in humans following exposure in water-damaged buildings. Arch Environ Health (2003) 58(7):410–20. 10.1080/00039896.2003.11879142 15143854

[35] Campbell AW Thrasher JD Madison RA Vojdani A Gray MR Johnson A. Neural autoantibodies and neurophysiologic abnormalities in patients exposed to molds in water-damaged buildings. Arch Environ Health (2003) 58(8):464–74. 10.3200/AEOH.58.8.464-474 15259425

[36] Bourque PR Chardon JW Massie R. Autoimmune peripheral neuropathies. Clin Chim Acta (2015) 449:37–42. 10.1016/j.cca.2015.02.039 25748038

[37] Vlachopoulou E Lahtela E Wennerstrom A Havulinna AS Salo P Perola M Evaluation of HLA-DRB1 imputation using a Finnish dataset. Tissue Antigens (2014) 83(5):350–5. 10.1111/tan.12343 24666112

[38] Rosenblum Lichtenstein JH Hsu YH Gavin IM Donaghey TC Molina RM Thompson KJ Environmental mold and mycotoxin exposures elicit specific cytokine and chemokine responses. PLoS One (2015) 10(5):e0126926. 10.1371/journal.pone.0126926 26010737 PMC4444319

[39] Fukutomi Y Tanimoto H Yasueda H Taniguchi M. Serological diagnosis of allergic bronchopulmonary mycosis: progress and challenges. Allergol Int (2016) 65(1):30–6. 10.1016/j.alit.2015.08.004 26740298

[40] Page ID Richardson MD Denning DW. Comparison of six *Aspergillus*-specific IgG assays for the diagnosis of chronic pulmonary aspergillosis (CPA). J Infect (2016) 72(2):240–9. 10.1016/j.jinf.2015.11.003 26680697

[41] Mirkovic B Lavelle GM Azim AA Helma K Gargoum FS Molloy K The basophil surface marker CD203c identifies *Aspergillus* species sensitization in patients with cystic fibrosis. J Allergy Clin Immunol (2016) 137(2):436–43.e9. 10.1016/j.jaci.2015.07.045 26388311

[42] Gernez Y Waters J Tirouvanziam R Herzenberg L Moss R Basophil activation test determination of CD63 combined with CD203c is not superior to CD203c alone in identifying allergic bronchopulmonary aspergillosis in cystic fibrosis. J Allergy Clin Immunol (2016) 138(4):1195–6. 10.1016/j.jaci.2016.04.002 27215492

[43] Edvardsson B Stenberg B Bergdahl J Eriksson N Linden G Widman L. Medical and social prognoses of non-specific building-related symptoms (sick building syndrome): a follow-up study of patients previously referred to hospital. Int Arch Occup Environ Health (2008) 81(7):805–12. 10.1007/s00420-007-0267-z 17924130

[44] Hope J. A review of the mechanism of injury and treatment approaches for illness resulting from exposure to water-damaged buildings, mold, and mycotoxins. ScientificWorldJournal (2013) 2013:767482. 10.1155/2013/767482 23710148 PMC3654247

[45] Rea WJ Pan Y Johnson AR. Clearing of toxic volatile hydrocarbons from humans. Bol Asoc Med P R (1991) 83(7):321–4. 1817511

[46] Rea WJ Pan Y Griffiths B. The treatment of patients with mycotoxin-induced disease. Toxicol Ind Health (2009) 25(9–10):711–4. 10.1177/0748233709348281 19854821

[47] Rea WJ Restrepo C Pan Y. Terpenes and terpenoids in chemical sensitivity. Altern Ther Health Med (2015) 21(4):12–7. 26030111

[48] Rea WJ Ross GH Johnson AR Smiley RE Fenyes EJ. Chemical sensitivity in physicians. Bol Asoc Med P R (1991) 83(9):383–8. 1807271

[49] Bavarsad Shahripour R Harrigan MR Alexandrov AV *N*-acetylcysteine (NAC) in neurological disorders: mechanisms of action and therapeutic opportunities. Brain Behav (2014) 4(2):108–22. 10.1002/brb3.208 24683506 PMC3967529

